# Associations Between Core Symptoms of Attention Deficit Hyperactivity Disorder and Both Binge and Restrictive Eating

**DOI:** 10.3389/fpsyt.2018.00103

**Published:** 2018-03-29

**Authors:** Panagiota Kaisari, Colin T. Dourish, Pia Rotshtein, Suzanne Higgs

**Affiliations:** ^1^School of Psychology, University of Birmingham, Birmingham, United Kingdom; ^2^P1vital, Wallingford, United Kingdom

**Keywords:** attention deficit hyperactivity disorder, inattention, binge eating, restrictive eating, implications for therapy

## Abstract

**Introduction:**

It is unclear whether core symptoms of attention deficit hyperactivity disorder (ADHD) relate to specific types of disordered eating and little is known about the mediating mechanisms. We investigated associations between core symptoms of ADHD and binge/disinhibited eating and restrictive eating behavior and assessed whether negative mood and/or deficits in awareness and reliance on internal hunger/satiety cues mediate these relationships.

**Methods:**

In two independent studies, we used a dimensional approach to study ADHD and disordered eating. In Study 1, a community-based sample of 237 adults (72.6% female, 18–60 years [M = 26.8, SE = 0.6]) completed an online questionnaire, assessing eating attitudes/behaviors, negative mood, awareness, and reliance on internal hunger/satiety cues and ADHD symptomatology. In Study 2, 142 students (80.3% female, 18–32 years [M = 19.3, SE = 0.1]) were recruited to complete the same questionnaires and complete tasks assessing interoceptive sensitivity and impulsivity in the laboratory.

**Results:**

In each study, core symptoms of ADHD correlated positively with both binge/disinhibited and restrictive eating and negative mood mediated the relationships. Deficits in awareness and reliance on internal hunger/satiety signals also mediated the association between inattentive symptoms of ADHD and disordered eating, especially binge/disinhibited eating. The results from both studies demonstrated that inattentive symptoms of ADHD were also directly related to binge/disinhibited eating behavior, while accounting for the indirect pathways of association *via* negative mood and awareness and reliance on internal hunger/satiety signals.

**Conclusion:**

This research provides evidence that core symptoms of ADHD are associated with both binge/disinhibited eating and restrictive eating behavior. Further investigation of the role of inattentive symptoms of ADHD in disordered eating may be helpful in developing novel treatments for both ADHD and binge eating.

## Introduction

Attention deficit hyperactivity disorder (ADHD) is one of the most debilitating childhood disorders, defined by age inappropriate impulsiveness, inattention, and hyperactivity (1), with symptoms of the disorder persisting into adulthood in approximately 75% of cases (2). ADHD is linked with a number of adverse outcomes (3), and accumulating evidence suggests that there is a strong association between ADHD and eating disorders (EDs)/disordered eating (4–7). Findings from previous studies suggest that specific symptoms of ADHD, mainly in the areas of attention (i.e., inattentive symptoms) and behavior regulation (i.e., impulsivity symptoms) may relate to disordered eating (8–11). Thus, impulsivity symptoms of ADHD have been consistently, positively associated with bulimic and binge eating behavior (12–14). However, the evidence to date for an association between inattentive symptoms of ADHD and EDs/disordered eating is mixed. For example, Seitz et al. (15) reported that in females seeking treatment for bulimia nervosa (BN), the severity of BN was best predicted by inattentive symptoms, rather than impulsivity or hyperactivity symptoms of ADHD. In contrast, Yilmaz et al. (11) found that a combination of “inattentive and hyperactive/impulsive” symptoms in children was the best predictor of higher eating disorder symptomatology during late adolescence. Limited evidence also exists for an association between hyperactive symptoms of ADHD and EDs/disordered eating, particularly restrictive eating behavior in men [for a review see Ref. (4)]. Therefore, further research is required to assess whether core symptoms of ADHD, namely inattentive, hyperactive, and impulsive symptoms, relate to specific subtypes of disordered eating (e.g., binge/disinhibited eating and/or restrictive eating behavior).

Disordered eating refers to patterns of eating behavior that deviate from normal, but do not meet all the criteria for a clinical diagnosis of an ED. Although the recorded ED prevalence rate is around 0.5–3%, depending on the specific ED diagnosis, disordered eating symptomatology in the general population has been found to be as high as 12% (16). This is a concern not only because of the associated psychopathology, but also because disordered eating is a risk factor for full-syndrome eating disorders (17). It is, therefore, important to explore risk factors for disordered eating, such as ADHD symptomatology, in the general population. This approach is predicated on the assumption that personality factors and symptoms of a disorder are best conceptualized dimensionally and occur with normal variation in the population (18). This dimensional approach ensures that potential confounds associated with clinical research in patients (e.g., medication status) can be minimized. Furthermore, the National Institute of Mental Health Research Domain Criteria Initiative (RDoC) encourages research on dimensions of observable behavior rather than a categorical, symptom-based approach to the study of mental health (19).

It is plausible that mood changes associated with depression, anxiety, and stress mediate the relationship between ADHD and risk for disordered eating. Indeed, several studies have reported that depression and anxiety are the most frequently reported psychiatric comorbidities in ADHD patients (20–23). A strong positive association between ADHD symptoms in adulthood and levels of self-perceived stress has also been reported (24). Furthermore, EDs, including binge/purging type (e.g., BN) and restrictive type [e.g., anorexia nervosa (AN)], are often comorbid with anxiety, depression, and/or mood disorders (25–27). Thus, it is possible that disordered eating may not be directly related to ADHD, and may be mediated (at least in part) by comorbid anxiety disorders and mood disorders (28–30). It is conceivable that individuals with ADHD could engage in disordered eating in an attempt to deal with frustrations and negative affect caused by the outcomes of attention difficulties (e.g., poor work performance) and/or impulsive responses at the cost of planned goals (31). Taken together, this evidence indicates that core symptoms of ADHD may relate to disordered eating, including both binge/disinhibited and restrictive eating indirectly *via* negative mood.

Another possibility is that the relationship between ADHD and disordered eating is mediated by lack of awareness of internal signals of hunger and satiety, which could contribute to a disturbed pattern of eating behavior. For instance, individuals with high inattentive symptoms of ADHD may forget about eating when engaged in other activities, leading to subsequent overeating or lack of control over eating (32). A lack of awareness and knowledge of internal signals of hunger and satiety may also contribute to restrictive eating pathology if external control, such as restricting food intake is used to override internal signals to adhere to a culturally imposed thin-ideal stereotype (33). Hence, core symptoms of ADHD may contribute to both binge/disinhibited eating and restrictive eating indirectly by a lack of awareness of internal signs of hunger and satiety.

Despite this evidence to the best of our knowledge no mediational studies have been conducted to date to assess whether negative mood and/or awareness and reliance on internal hunger/satiety signs provide pathways of association between ADHD symptomatology and disordered eating. The current research investigated the potential associations between core symptoms of ADHD and disordered eating, including both binge/disinhibited eating and restrictive eating behavior, and examined whether negative mood and/or deficits in awareness and reliance on internal hunger/satiety cues might mediate these relationships. Two studies were conducted: Study 1 was an online study, which investigated the hypothesized mediational pathways of association between hyperactivity/impulsivity and inattentive symptoms of ADHD and disordered eating *via* negative mood and/or deficits in awareness and reliance on internal hunger/satiety cues. Study 2 was a laboratory study, which investigated whether the findings from Study 1 could be replicated in an independent sample, and assessed the specific contributions *(if any)* of hyperactivity and impulsivity to disordered eating.

## Study 1

The primary aims of Study 1 were: (i) to investigate the associations between core symptoms of ADHD and disordered eating, including both binge/disinhibited eating and restrictive eating behavior, and (ii) to test whether negative mood and/or and deficits in awareness and reliance on internal hunger/satiety cues might mediate these relationships, controlling important potential confounders (e.g., gender, BMI, and ADHD medication). The moderating effect of age, gender, BMI, and ADHD medication in any relationship between core symptoms of ADHD and disordered eating was also investigated.

## Materials and Methods

### Participants and Procedure

Participants were recruited from the general population, through social media (e.g., Facebook), postings on web sites, such as ADHD support groups, and advertisements at the University of Birmingham, UK. Adult men and women, aged 18–60 years, differing in severity of ADHD symptoms were eligible for this study. Individuals receiving medication for ADHD treatment were not excluded, but medication status was controlled for in the analysis. Participants were required to be fluent in English. Since no mediational studies have been conducted to date to investigate the role of negative mood and/or awareness and reliance on internal hunger/satiety signals in mediating the relationship between symptoms of ADHD and disordered eating behavior, a formal power calculation was not conducted. However, Fritz and MacKinnon (34) report that for medium size pathways of association, a sample size of 71 participants would be adequate for 0.8 Power when using a bias-corrected bootstrap method, as we used in this study. From November 2015 to March 2016, a total of 265 individuals completed an online survey. All participants provided online informed consent before participation. After informed consent was obtained, participants were directed to a URL to complete the online survey. The survey took approximately 45 min to complete. All participants were informed that they could enter a prize draw to win a £50 gift voucher for their participation. The protocol was approved by the University of Birmingham Research Ethics Committee.

### Measures

#### Data Quality

Due to the self-administered nature of online surveys, measurement error is a common problem. In the present study, certain techniques were used to allow a quality assessment of the collected responses. These are described in brief below.

##### Trap Questions

Trap questions (e.g., for data quality purposes, please select 4) were used for every 50–100 survey items (3 in total) (35) to identify participants who were not paying sufficient attention to instruction and not providing consistent responses.

##### Self-Report of Study Engagement

At the end of the survey, participants were asked to self-report the quality of the data they provided (35). Specifically, they were asked to respond to the following question: “Finally, it is vital to our study that we only include responses from people who devoted their full attention to this study. In your honest opinion, should we use your data?” It was emphasized that participants could still enter the prize draw to win the gift voucher for their participation regardless of how they responded to the question.

To minimize measurement error, which may lead to biased results it was decided *a priori* that responses from participants who answered all three of the trap questions incorrectly would be removed from any further analysis. A similar approach was followed for participants who responded negatively to the self-report measure of study engagement.

### Demographics

Demographics, including age, sex, ethnicity, education level, and professional status were recorded. Body mass index (BMI; kg/m^2^) was calculated based on self-reported measures of height and body weight. A proxy measure of socio-economic status (SES) was calculated based on the participants’ responses about their highest education level completed and their current profession. The education level was collapsed into six categories, including some secondary or high school education (including O Levels and GCSEs); A levels, high school diploma, high school certificate, etc.; vocational qualifications; University or college graduate; Master’s degree, post-graduate certificate or diploma, and professional or doctoral degree. Profession status was categorized as: unemployed; student; semi-skilled or unskilled manual worker; skilled manual worker; supervisor or clerical, junior managerial, administrative or professional; intermediate managerial, administrative or professional; higher managerial, administrative or professional. A 6- and 7-point scale was used to score the education and profession variables, respectively, with higher scores representing higher education level completed and higher managerial, administrative, or professional status. The SES was calculated as the mean score of the two variables.

### Current Symptoms of ADHD

#### The Conners’ Adult ADHD Rating Scale-Self-Report Screening Version (CAARS-S: SV)

The Conners’ Adult ADHD Rating Scale-Self Report Screening Version (36) was used to assess current ADHD symptoms. The CAARS is a suitable instrument for evaluating ADHD symptoms in adults (37) and utilizes a 4-point format in which respondents are asked to rate items pertaining to their problems. The CAARS-S: SV has 30 items that assess ADHD symptoms according to the fourth edition of the diagnostic and statistical manual of mental disorders. The subscales derived from the questionnaire are: (a) Inattentive Symptoms (9 items); (b) Hyperactive-Impulsive Symptoms (9 items); (c) Total ADHD Symptoms; (d) ADHD Index (12 items). In this study, the Cronbach’s alpha was 0.94, 0.89, 0.95, and 0.88 for the “Inattentive Symptoms,” “Hyperactive-Impulsive Symptoms,” “Total ADHD Symptoms,” and “ADHD Index” subscales, respectively.

Participants were also asked to report whether they had been diagnosed with ADHD by a healthcare professional. If they answered in the affirmative, they were asked at what age the diagnosis took place, and if they were currently taking medication as part of their treatment protocol or if they received medication during the previous year. If medication was taken, the participant was asked to report the type of medication (e.g., stimulant). ADHD medication use was coded as a dichotomous variable. Other psychological/psychiatric comorbidities were categorized as present, including any other psychological/psychiatric disorders diagnosed by a healthcare professional apart from ADHD and/or anxiety, depression, and eating disorders, or not present.

### Disordered Eating

Several established measures of disordered eating were included in the survey:

*The Dutch Eating Behavior Questionnaire (DEBQ)* (38), a 33-item self-report questionnaire, was used to assess three aspects of eating behavior: “emotional eating” (Factor I—13 items), “external eating” (Factor II—10 items), and “dietary restraint” (Factor III—10 items). These subscales have been shown to have good reliability and validity (38). In this study, the Cronbach’s alpha was 0.96, 0.89, and 0.93 for the “emotional eating,” “external eating,” and “dietary restraint” subscales, respectively.
*The Loss of Control over Eating Scale (LOCES)* (39), short version, a 7-item scale, was used to assess subjective perceptions of being compelled to eat or unable to resist or stop eating, resulting in initiating eating when not intended, and/or eating more than originally intended, and/or difficulty stopping eating. The 7-item LOCES utilizes a 5-point scale and responders are asked to rate how often in the past 4 weeks they experienced each one of the 7 specified items during a time when they were eating (*e.g., my eating felt like a ball rolling down a hill that just kept going and going*). The tool has been shown to have good test–retest reliability (*r* = 0.82, *p* < 0.001) and validity (Cronbach’s alpha = 0.93). In this study, the Cronbach’s alpha was 0.95.
*The Binge Eating Scale (BES)* (40), a 16-item self-report questionnaire, was used to assess binge eating pathology. The BES yields a continuous measure of binge eating pathology of 0–46. The BES has good test–retest reliability (*r* = 0.87, *p* < 0.001) and moderate associations with binge eating severity as measured by food records (*r* = 0.20–0.40, *p* < 0.05) (41). The Cronbach’s alpha in this study was 0.92.
*The Bulimic Investigatory Test, Edinburgh (BITE)* (42), is a 33-item self-report questionnaire, designed as an objective screening test to identify subjects with bulimic symptoms. The BITE consists of two subscales: the symptoms scale (30 items) and the severity scale (3 items). Results from the symptoms scale were analyzed in this study. The symptoms scale score ranges from 0 to 30. Internal consistency of the BITE symptom scale is high (0.96) (42). In this study, the Cronbach’s alpha was 0.92.
*The Eating Attitudes Test (EAT-26)* (43), a 26-item self-report questionnaire, has been widely applied as an index of the symptoms of AN. The EAT-26 comprises three subscales, Dieting (Factor I—13 items), Bulimia and Food preoccupation (Factor II—6 items), and Oral Control (Factor III—7 items), which are summed to obtain a total score. The tool has been used in both clinical and non-clinical settings and has been found to have strong psychometric properties (43–45). The Cronbach’s alpha measures in this study were 0.88, 0.87, 0.82, and 0.66 for the EAT Total score, and the Dieting, Bulimia, and Oral Control subscales, respectively.

### Awareness and Reliance on Internal Hunger/Satiety Cues

#### The Reliance on Internal Hunger/Satiety Cues Subscale

The Reliance on Internal Hunger/Satiety Cues subscale of the 21-item Intuitive Eating Scale (IES) (46) was used to assess awareness and use of internal hunger/satiety cues to guide one’s eating behavior. The Reliance on Internal Hunger/Satiety Cues subscale comprises six items (e.g., “I can tell when I’m slightly hungry”), and item responses are rated on a scale that ranges from 1 (strongly disagree) to 5 (strongly agree). After appropriate items are reverse-scored, item responses are averaged to arrive at a total score. Higher total scores correspond with higher levels of awareness and use of internal hunger/satiety cues to determine one’s eating behavior. The Reliance on Internal Hunger/Satiety Cues subscale has been found to be positively correlated with interoceptive sensitivity, as assessed by the heartbeat perception task (47). In this study, the Cronbach’s alpha was 0.85.

### Negative Mood

Negative mood was modeled as a latent variable comprising three emotional factors associated with anxiety and depression and with stress perception: anxiety and depression levels were assessed by the 14-item Hospital Anxiety and Depression Scale (HADS) (48). The HADS is a valid tool to measure anxiety and depression levels in the general population and performs well in screening for the separate dimensions of anxiety and depression (49). The Cronbach’s alpha in this study was 0.85 and 0.82 for the anxiety and depression subscales, respectively. To assess perception of stress, participants were asked to complete the 10-item Perceived Stress Scale (PSS) (50). The PSS has consistently shown high internal validity (Cronbach’s alpha > 0.70) and good test–retest reliability (Rho > 0.70) (51, 52). In this study, the Cronbach’s alpha was 0.90.

### Alcohol and Drug Use

Alcohol abuse was assessed by the Short Michigan Alcohol Screening Test (SMAST) (53). Evaluation data indicate that it is an effective diagnostic instrument, and does not have a tendency for false positives. In this study, the Cronbach’s alpha was 0.73. Drug abuse was assessed by the Drug Abuse Screening Test-10 (DAST-10) (54), a 10-item self-report scale that consists of items that parallel those of the Michigan Alcoholism Screening Test (MAST). The DAST-10 has exhibited valid psychometric properties for use as a clinical or research tool in a variety of populations (55). In this study, the Cronbach’s alpha was 0.68. Twelve participants had missing data; therefore, the available sample size for the internal consistency reliability analysis was limited to 225 participants.

## Analysis Plan

### Data Reduction

To condense the information contained in the disordered eating variables into a smaller set of new composite dimensions, with a minimum (min) loss of information, a principal component analysis (PCA) with varimax rotation was applied, using SPSS. PCA revealed that measures of disordered eating loaded onto two components (for component loadings, see Table S1, Data Sheet S1 in Supplementary Material). Together the components explained 73% of the variance observed. The first component (eigenvalue = 5.03), which accounted for 56% of the variance, was labeled “Binge/Disinhibited Eating.” The second component (eigenvalue = 1.56) accounted for a further 17% of the variance and was labeled “Restrictive Eating.” A high score on the “Binge/Disinhibited Eating” component is related to a pattern of behavior that involves a tendency toward overeating and eating opportunistically. This can include eating in response to negative affect, overeating in response to the palatability of food and not being able to resist temptation to eat despite negative consequences. The mean composite score for this component was 31.4 with a SD of 21.3. A high score on the “Restrictive Eating” component is related to a pattern of behavior to limit food intake as a means of controlling body weight. The mean composite score for this component was 11.9 with a SD of 9.7.

### Negative Mood

A composite score reflecting an overall psychological distress was calculated for the three emotional factors associated with anxiety and depression (HADS-Anxiety and HADS-Depression), and stress perception (PSS) using PCA. The extracted component accounted for 74% of the variance in the three emotional scales, and all three loaded strongly on this factor (loadings ranged from 0.806 to 0.897). The mean composite score was 35.7 with a SD of 14.8.

### Models

Mediation and moderation effects were examined using PROCESS for SPSS (56). Specifically, Model 4 with multiple parallel mediators *(negative mood, awareness, and reliance on internal hunger/satiety cues)* was tested to investigate the hypothesized relationships between core symptoms of ADHD and both binge/disinhibited and restrictive eating behavior. Indirect (i.e., mediating) effects were evaluated with 95% bias-corrected confidence intervals based on 10,000 bootstrap samples. When the confidence interval does not contain zero, this indicates that the indirect effect can be considered statistically significant (56). To assess whether age, gender, BMI, or ADHD medication moderates any relationship between core symptoms of ADHD and disordered eating, Model 5 was tested. Moderation is found if any of the tested interactions (e.g., inattentive symptoms of ADHD × gender) is statistically significant (*p* < 0.005). Continuous variables were mean centered as recommended by Howell (57). All analysis was conducted using SPSS 22.0 software (IBM Corp., Armonk, NY, USA).

### Covariates

Based on their potential for having associations with disordered eating and/or ADHD, it was decided *a priori* to include eight variables in the statistical analyses: age, gender, BMI, SES, current ADHD medication use, other psychological/psychiatric comorbidities, alcohol, and drug use. Alcohol and drug abuse were coded as continuous variables.

## Results

### Participants

A total of 265 individuals completed the online survey. Nine participants (3.4%) categorized themselves as “other” gender, and due to the small representation of this group in our sample population, these individuals were excluded from analyses. Further, 19 participants were excluded because of: (1) failure to meet inclusion criteria (*n* = 1; aged >60 years old); (2) non-existent or very incomplete responses (≥80% of missing data) (*n* = 11); or (3) low quality responses based on self-report measure of study engagement (*n* = 7). The final sample consisted of 237 individuals.

Participants’ age ranged between 18 and 60 years old, with a mean of 26.8 years (SE = 0.6). Most of the participants were females (72.6%) and were of White/White British (78.1%) ethnicity. Other ethnic groups included: 10.1% Asian/Asian British; 1.7% Black/African/Caribbean/Black British; 4.2% Mixed/multiple ethnic groups, and 5.9% reported Other ethnic group. The majority of the participants were European (57.4%) or North American (35.0%) residents, while smaller representation included Australia/New Zealand (3.4%), Asia (3.0%), South America (0.4%), and Other (0.8%). More than half of the participants were highly educated (55.3%); 30.4% were university or college graduates, 23.6% had completed a Master’s degree, post-graduate certificate, or diploma; and 1.3% had a professional or doctoral degree. Among the remaining participants: 30.8% had completed A Levels, High School Diploma, High School Certificate, etc.; 5.5% Vocational qualifications; 3.0% Some secondary or high school education (including O Levels and GCSEs); and 5.5% reported Other highest education completed. Most of the respondents were students (59.5%). Other professions reported were: higher managerial, administrative or professional (2.1%); intermediate managerial, administrative or professional (11.0%); supervisor or clerical, junior managerial, administrative or professional (9.7%); skilled manual worker (1.3%); semi-skilled or unskilled manual worker (3.4%); unemployed (7.6%). A wide range of BMIs (12.49–68.52 kg/m^2^) was represented, with a mean of 24.6 kg/m^2^ (SE = 0.4). Most of the participants, 127 (53.6%) had a BMI within the normal range; 53 (22.4%) were classified as overweight; 32 (12.5%) as obese; and 21 (8.9%) as underweight. Four participants had missing data and, therefore, their BMI could not be calculated.

### Data Quality

The vast majority of the responders (97.5%) answered all of the three trap questions correctly, suggesting that they were paying close attention to directions.

### Current ADHD Symptoms

Table 1 presents the mean and SE, as well as the minimum (min) and the maximum (max) scores for the four subscales derived from the CAARS: S-SV. Of the 237 individuals, 79 (33.3%) (males = 20; females = 59) had received a diagnosis of ADHD and 59 (24.9%) were currently being treated with medication (stimulant = 50; non-stimulant = 9). The mean reported age of ADHD diagnosis was 20.06 years (SE = 1.18); 35.4% reported being diagnosed before the age of 18 years and 64.6% reported being diagnosed in adult life.

**Table 1:** Mean, SE, min, and max scores on the four subscales derived from the CAARS: S-SV.

	Total sample (*n* = 237)			
Current attention deficit hyperactivity disorder (ADHD) symptoms	Mean	SE	Min	Max
Inattentive symptoms (0–27)	13.80	0.50	1	27
Hyperactive-impulsive symptoms (0–27)	10.18	0.41	0	27
Total ADHD symptoms (0–54)	23.97	0.85	3	54
ADHD index (0–36)	16.67	0.52	3	34

### Multiple Mediation Analyzes

**Figure 1:**
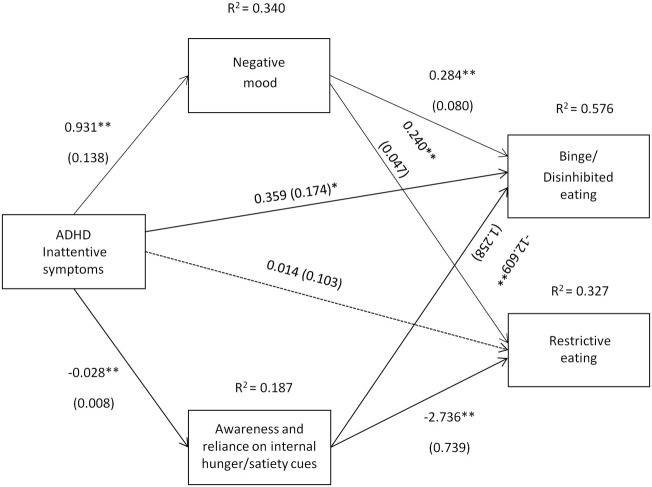
Attention deficit hyperactivity disorder (ADHD) inattentive symptoms [Conners’ Adult ADHD Rating Scale-Self-Report Screening Version, “Inattentive Symptoms” subscale] and their relation to binge/disinhibited eating and restrictive eating. Test for mediation through negative mood (composite measure) and awareness and reliance on internal hunger/satiety cues (self-report measure). Estimates are unstandardized regression coefficients; *R*^2^ is their explained variance for the consequents; numbers in parentheses are bootstrapped SEs. All analyses controlled for age, gender, BMI, socio-economic status, other comorbid psychiatric disorders, current ADHD medication use, and alcohol use. Simple arrows: significant path coefficients, dotted arrows: non-significant path coefficients.*P < 0.05; **P < 0.01.

**Figure 2:**
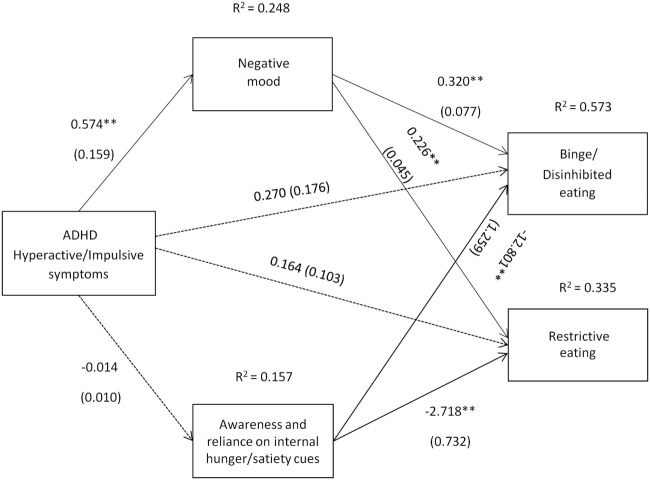
Attention deficit hyperactivity disorder (ADHD) hyperactive/impulsive symptoms [Conners’ Adult ADHD Rating Scale-Self-Report Screening Version, “Hyper active-Impulsive” symptoms subscale] and their relation to binge/disinhibited eating and restrictive eating. Test for mediation through negative mood (composite measure) and awareness and reliance on internal hunger/satiety cues (self-report measure). Estimates are unstandardized regression coefficients; *R*^2^ is their explained variance for the consequents; numbers in parentheses are bootstrapped SEs. All analyses controlled for age, gender, BMI, socio-economic status, other comorbid psychiatric disorders, current ADHD medication use, and alcohol use. Simple arrows: significant path coefficients, dotted arrows: non-significant path coefficients.*P < 0.05; **P < 0.01.

The path coefficients, SEs, and other statistics pertinent to the models are superimposed on the statistical diagrams in Figures 1 and 2. Age, gender, BMI, SES, other comorbid psychiatric disorders, current ADHD medication use, and alcohol use were covariates in the models. Drug use was found to be significantly correlated with alcohol use (*r* = 0.33), and, therefore, was not included as an additional covariate.

Inattentive symptoms of ADHD predicted binge/disinhibited eating both directly (*c*′ = 0.359, *p* = 0.041) and indirectly through an effect on negative mood, a_1_b_1_ = 0.264 BCa CI [0.114, 0.456] and on awareness and reliance on internal hunger/satiety cues, a_2_b_2_ = 0.357 BCa CI [0.157, 0.602] (for details on all path coefficients see Figure 1). The mediating effect of negative mood was of similar strength to the mediating effect of awareness and reliance on internal hunger/satiety cues BCa CI [−0.378, 0.162]. Inattentive symptoms of ADHD also predicted restrictive eating, but only indirectly through an effect on negative mood, a_1_b_1_ = 0.223 BCa CI [0.130, 0.345] and on awareness and reliance on internal hunger/satiety cues, a_2_b_2_ = 0.077 BCa CI [0.022, 0.178] (for details on all path coefficients see Figure 1). The mediating effect of negative mood was stronger than the mediating effect of awareness and reliance on internal hunger/satiety cues BCa CI [0.023, 0.278].

Hyperactive/impulsive symptoms of ADHD also indirectly predicted binge/disinhibited eating through an effect on negative mood, a_1_b_1_ = 0.184 BCa CI [0.066, 0.351] (for details on all path coefficients see Figure 2). Hyperactive/impulsive symptoms of ADHD also predicted restrictive eating indirectly through an effect on negative mood, a_1_b_1_ = 0.130 BCa CI [0.059, 0.225] (for details on all path coefficients see Figure 2).

### Moderation Analyses

Conditional path analysis indicated that none of the relationships between core symptoms of ADHD and disordered eating were moderated by age, gender, BMI, or ADHD medication (*p_s_* > 0.005).

## Study 2

The aim of Study 2 was to investigate whether the findings from Study 1, which was conducted online, could be replicated in a well-defined clinical laboratory sample. In Study 2, we also investigated the specific contributions *(if any)* of hyperactivity and impulsivity to disordered eating. As the derived subscales of the CAARS-S:SV, do not provide separate scores for hyperactive and impulsive symptoms of ADHD, it is unclear whether both hyperactive and impulsive symptoms relate to binge/disinhibited and restrictive eating or whether specific ADHD symptoms relate to specific types of disordered eating. In Study 2, we tested the hypothesis that impulsivity symptoms specifically predict disordered eating behavior by including behavioral and self-reported measures of impulsivity and comparing a model using those measures as predictors of disordered eating with a model using the CAARS-S: SV “Hyperactive-Impulsive” Symptoms subscale (which measures both impulsivity and hyperactivity symptoms) as a predictor of disordered eating behavior. We hypothesized that both measures of impulsivity would correlate positively with the hyperactive/impulsive symptoms subscale of the CAARS-S: SV.

In Study 2, we assessed awareness and reliance on internal hunger/satiety cues *via* a self-report measure, as used in Study 1, but in addition used a performance-based measure of interoceptive sensitivity, the heartbeat perception task. The heartbeat perception task has been related to the ability to detect changes in other autonomically innervated organs, such as the activity of the stomach (58, 59), highlighting its role as an indicator of a generalized sensitivity for visceral processes in situations evoking interoceptive signals (60), such as during food deprivation and feeling hungry (61). We hypothesized that inattentive symptoms of ADHD would relate negatively to interoceptive sensitivity, and decreased interoceptive sensitivity would relate positively to disordered eating.

Overall, Study 2 aimed to: (i) investigate whether the findings from Study 1 can be replicated using a well-defined sample tested in the laboratory, (ii) investigate the specific contributions*(if any)* of hyperactivity and impulsivity to disordered eating, and (iii) investigate whether interoceptive sensitivity as assessed objectively *via* a heartbeat perception task provides another pathway of association between inattentive symptoms of ADHD and disordered eating as found in Study 1, when we used a self-report measure of awareness and reliance on internal hunger/satiety cues.

## Materials and Methods

### Participants and Procedure

Students from the University of Birmingham, UK were recruited in exchange for course credits or cash payment. From October 2016 to December 2016, a total 142 individuals completed the study. Participants were required to be fluent in English. The study was advertised as a two-part study, and participants had to complete an online questionnaire before a laboratory meeting was arranged to complete the second part of the study and receive full reimbursement for their participation. All participants provided online informed consent before completion of the online questionnaire. After informed consent was obtained, participants were directed to a URL to complete an online survey as described in Study 1. Following completion of the survey, a laboratory meeting was arranged and participants came to the School of Psychology, University of Birmingham to complete a battery of behavioral measures. Informed consent was also obtained before completion of the behavioral measures. The protocol was approved by the University of Birmingham Research Ethics Committee.

### Measures

#### Data Quality

Trap questions and a self-report measure of study engagement were used to enable a quality assessment of the collected responses, as described for Study 1.

#### Demographics

Demographics including age, sex, ethnicity, education level, and professional status were reported as in Study 1. Participants height and body weight were measured to calculate current BMI (kg/m^2^).

### Current Symptoms of ADHD

#### The Conners’ Adult ADHD Rating Scale-Self-Report Screening Version

The Conners’ Adult ADHD Rating Scale-Self-Report Screening Version (36) was used to assess current ADHD symptoms. In this study, the Cronbach’s alpha was 0.87, 0.84, 0.90, and 0.80 for the “Inattentive Symptoms,” “Hyperactive-Impulsive Symptoms,” “Total ADHD Symptoms,” and “ADHD Index” subscales, respectively.

### Questionnaire Measures

Questionnaire measures were the same as in Study 1. For the Cronbach’s alpha values in this study, see Table S2, Data Sheet S1 in Supplementary Material.

### Impulsivity

#### The Barratt Impulsivity Scale (BIS)

The Barratt Impulsivity Scale (BIS) (62), a 30-item self-report questionnaire, was used in Study 2 to assess impulsivity. Its psychometric properties have been determined in both clinical and non-clinical subjects (62, 63). In this study, the total score was used and the Cronbach’s alpha was 0.85.

#### Go/No-Go Task

Impulsivity was also assessed behaviorally using a go/no-go task assessing the ability to inhibit instigated, “prepotent” responses *(response inhibition)*. Participants were instructed to press the space bar in response to explicitly instructed “go” stimuli and withhold any response to “no-go” stimuli. Participants used their preferred hand to respond and complete two tasks: (a) a neutral go/no-go task and (b) a food go/no-go task (see Figure S1, Data Sheet S1 in Supplementary Material). The measure of interest was the number of commission errors (responses incorrectly made in “no-go” trials). Because commission errors are responses that occur when no response is required, they are suggested to reflect impulsivity. For details on the Go/No-Go task, see Data Sheet S1 in Supplementary Material.

#### Interoceptive Sensitivity: The Heartbeat Perception Task

For the heartbeat task, participants’ heart rate was monitored with a pulse oximeter (PulseOximeterOnline.com) to obtain their average heart rate. Following the well-validated Mental Tracking Method of Schandry (64), data were recorded for two time intervals (45, 60 s). While participants’ heart rate was monitored with a pulse oximeter, participants were asked to count silently their own heartbeats, without taking their pulse or attempting any other physical manipulation that could facilitate the detection of heartbeats. Participants were given verbal instructions when to start and when to stop counting. At the end of each time interval, participants were asked to verbally report how many heartbeats they counted. No feedback on the duration of the counting phases or the quality of their performance was given. Interoceptive sensitivity was measured according to the following transformation: 1/2 S (1–(|recorded heartbeats–counted heartbeats|)/recorded heartbeats). The heartbeat perception score varies between 0 and 1. The maximum (max) score of 1 indicates absolute accuracy of heartbeat perception.

## Analysis Plan

### Data Reduction

#### Disordered Eating

As in Study 1, composite scores were created for “Binge/Disinhibited Eating” and for “Restrictive Eating.” In this study, the mean composite score for “Binge/Disinhibited Eating” was 25.1 with a SD of 17.9 and the mean composite score for “Restrictive Eating” was 9.5 with a SD of 9.1.

#### Negative Mood

In this study, the extracted component accounted for 77% of the variance in the three emotional scales, and all three scales loaded strongly on this factor (range from 0.855 to 0.893). The mean composite score was 29.6 with a SD of 12.4.

#### Models

Mediation and moderation effects were examined using PROCESS for SPSS (56) (for details see Analysis Plan Study 1). To investigate whether both measures of impulsivity correlated positively with the hyperactivity/impulsivity symptoms subscale of the CAARS-S:SV as hypothesized, Spearman’s correlations for non-normally distributed variables were computed. Spearman’s correlations were also computed to assess whether inattentive symptoms of ADHD correlated negatively to interoceptive sensitivity, and decreased interoceptive sensitivity correlated positively to disordered eating.

#### Covariates

As in Study 1, the following variables were included in the statistical analyses: age, gender, and BMI. Current ADHD medication use and other psychological/psychiatric comorbidities were not included in the statistical analyses, as none of the participants reported either use of ADHD medication or any psychological/psychiatric comorbidity. In addition, SES was not included in the statistical analysis, as all participants were students. The internal consistency for the SMAST questionnaire was poor in the present study (Cronbach’s alpha = 0.51). In addition, only 7 (4.9%) participants in total reported a score equal to or greater than 3, which has been suggested to indicate a borderline alcohol problem or potential alcohol abuse. Therefore, alcohol use was not included as a covariate in the statistical analysis.

## Results

### Participants

A total of 142 participants completed the study. Age ranged from 18 to 32 years old, with a mean of 19.3 years (SE = 0.1). Most of the participants were females (80.3%) and were of White/White British (68.3%) ethnicity. Other ethnic groups included: 19.0% Asian/Asian British; 7.0% Black/African/Caribbean/Black British; 2.8% Mixed/multiple ethnic groups; and 2.8% reported Other ethnic group. BMIs ranged between 14.7–34.4 kg/m^2^, with a mean of 21.4 kg/m^2^ (SE = 0.3). The majority of the participants, 93 (65.5%) had a BMI within the normal range; 26 (18.3%) were classified as underweight; 20 (14.1%) as overweight; and 2 (1.4%) as obese. One participant had missing data and, therefore, their BMI could not be calculated.

### Data Quality

The vast majority of the responders (98.6%) answered all of the three trap questions correctly, suggesting that they were paying close attention to directions.

### Current ADHD Symptoms

Table 2 presents the mean and SE, as well as the minimum (min) and the maximum (max) scores for the four subscales derived from the CAARS: S-SV.

**Table 2:** Mean, SE, min, and max scores on the four subscales derived from the CAARS: S-SV.

	Total sample (*n* = 142)			
Current attention deficit hyperactivity disorder (ADHD) symptoms	Mean	SE	Min	Max
Inattentive symptoms (0–27)	9.23	0.43	0	24
Hyperactive-impulsive symptoms (0–27)	7.25	0.40	0	22
Total ADHD symptoms (0–54)	16.49	0.75	0	45
ADHD index (0–36)	11.71	0.47	2	31

### Multiple Mediation Analyzes

#### Aim 1

To investigate whether the findings from Study 1 can be replicated using a well-defined sample tested in the laboratory.

**Figure 3:**
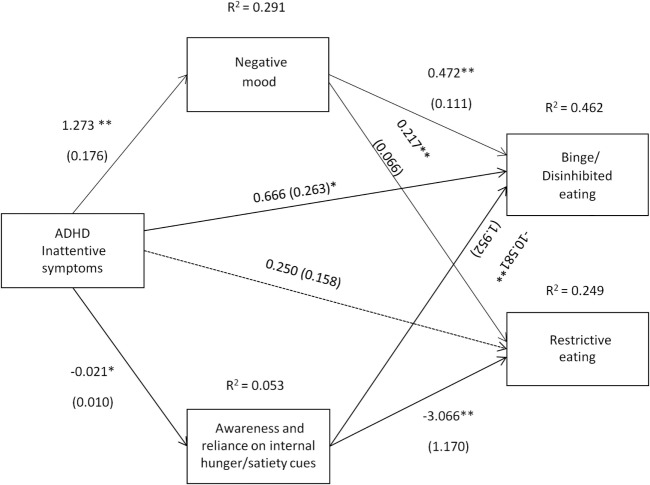
Attention deficit hyperactivity disorder (ADHD) inattentive symptoms [Conners’ Adult ADHD Rating Scale-Self-Report Screening Version, “Inattentive Symptoms” subscale] and their relation to binge/disinhibited eating and restrictive eating. Test for mediation through negative mood (composite measure) and awareness and reliance on internal hunger/satiety cues (self-report measure). Estimates are unstandardized regression coefficients; *R*^2^ is their explained variance for the consequents; numbers in parentheses are bootstrapped SEs. All analyses controlled for age, gender, and BMI. Simple arrows: significant path coefficients, dotted arrows: non-significant path coefficients.*P < 0.05; **P < 0.01.

**Figure 4:**
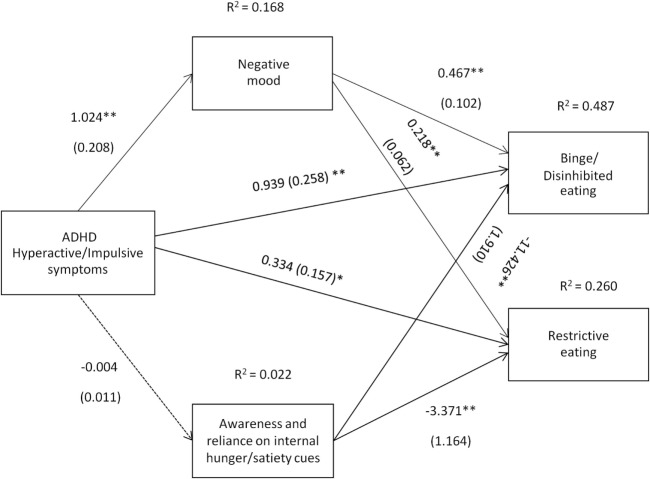
Attention deficit hyperactivity disorder (ADHD) hyperactive/impulsive symptoms [Conners’ Adult ADHD Rating Scale-Self-Report Screening Version, “Hyperactive-Impulsive” subscale] and their relation to binge/disinhibited eating and restrictive eating. Test for mediation through negative mood (composite measure) and awareness and reliance on internal hunger/satiety cues (self-report measure). Estimates are unstandardized regression coefficients; *R*^2^ is their explained variance for the consequents; numbers in parentheses are bootstrapped SEs. All analyses controlled for age, gender, and BMI. Simple arrows: significant path coefficients, dotted arrows: non-significant path coefficients.*P < 0.05; **P < 0.01.

The path coefficients, SEs, and other statistics pertinent to the models are superimposed on the statistical diagrams in Figures 3 and 4. Age, gender, and BMI were covariates in the models.

Inattentive symptoms of ADHD predicted binge/disinhibited eating both directly (*cI* = 0.666, *p* = 0.012) and indirectly through an effect on negative mood, a_1_b_1_ = 0.600 BCa CI [0.299, 1.013] and on awareness and reliance on internal hunger/satiety cues, a_2_b_2_ = 0.227 BCa CI [0.012, 0.544] (for details on all path coefficients see Figure 3). The mediating effect of negative mood was of similar strength to the mediating effect of awareness and reliance on internal hunger/satiety cues BCa CI [−0.016, 0.770]. Inattentive symptoms of ADHD predicted also restrictive eating, but only indirectly through an effect on negative mood, a_1_b_1_ = 0.276 BCa CI [0.117, 0.490]. Although increased inattentive symptoms of ADHD predicted lower levels of awareness and reliance on internal hunger/satiety cues (a_2_ = −0.021), and lower levels of awareness and reliance on internal hunger/satiety cues predicted higher restrictive eating (b_2_ = −3.066), a bias-corrected bootstrap confidence interval for the indirect effect (a_1_b_1_ = 0.066) based on 10,000 bootstrap samples was below zero (−0.003 to 0.227), suggesting that the mediating effect was not significant (for details on all path coefficients see Figure 3).

Hyperactive/impulsive symptoms of ADHD predicted binge/disinhibited eating both directly (*c*′ = 0.939, *p* < 0.01) and indirectly through an effect on negative mood, a_1_b_1_ = 0.478 BCa CI [0.243, 0.848] (for details on all path coefficients see Figure 4). Hyperactive/impulsive symptoms of ADHD also predicted restrictive eating both directly (*c*′ = 0.334, *p* = 0.036) and indirectly through an effect on negative mood, a_1_b_1_ = 0.223 BCa CI [0.110, 0.406] (for details on all path coefficients see Figure 4).

### Moderation Analyzes

Conditional path analysis indicated that none of the relationships between core symptoms of ADHD and disordered eating were moderated by age, gender, or BMI (*p_s_* > 0.005).

#### Aim 2

To investigate the specific contributions *(if any)* of hyperactivity and impulsivity to disordered eating.

**Figure 5:**
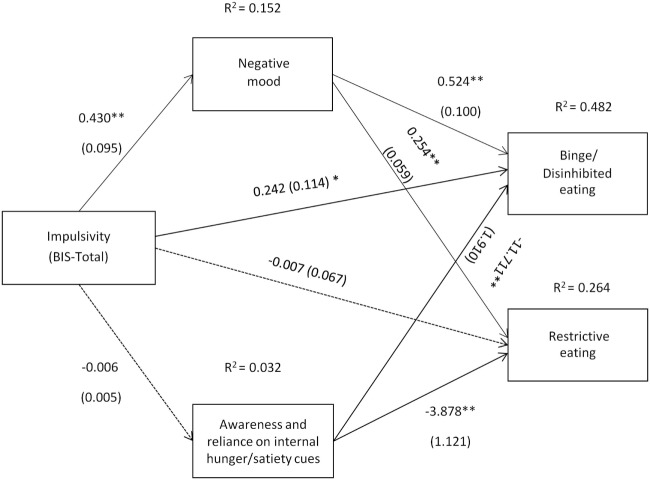
Impulsivity symptoms (Barratt Impulsivity Scale total score) and their relation to binge/disinhibited eating and restrictive eating. Test for mediation through negative mood (composite measure) and awareness and reliance on internal hunger/satiety cues (self-report measure). Estimates are unstandardized regression coefficients; *R*^2^ is their explained variance for the consequents; numbers in parentheses are bootstrapped SEs. All analyses controlled for age, gender, and BMI. Simple arrows: significant path coefficients, dotted arrows: non-significant path coefficients.*P < 0.05; **P < 0.01.

As hypothesized, hyperactivity/impulsivity symptoms of ADHD, assessed by the hyperactive-impulsive subscale of the CAARS-S: SV, were positively correlated with self-reported levels of impulsivity (*r* = 0.54; *p_s_* < 0.001), assessed by the BIS. Contrary to our hypothesis, hyperactivity/impulsivity symptoms of ADHD, were not significantly correlated with either the number of commission errors on the neutral no-go trials (*r* = 0.13; *p_s_* = 0.13) or the food no-go trials (*r* = 0.14; *p_s_* = 0.11), although the correlation coefficients are suggestive of positive associations. Therefore, to investigate the specific contributions *(if any)* of hyperactivity and impulsivity to disordered eating, we tested the same model as shown in Figure 4, but this time we used impulsivity as assessed by the BIS as the predictor variable (see Figure 5).

As shown in Figure 5, a similar pattern of results as those presented in Figure 4 was observed when impulsivity assessed by the BIS was used as a predictor variable. However, although there was evidence that impulsivity was associated with binge/disinhibited eating independent of an effect on mood (*c*′ = 0.242, *p* = 0.035), there was no evidence of a direct effect of impulsivity on restrictive eating (*c*′ = −0.007, *p* = 0.915).

#### Aim 3

To investigate whether interoceptive sensitivity mediates the association between inattentive symptoms of ADHD and disordered eating.

The mean score of interoceptive sensitivity was 0.71 (SE = 0.02). One participant performed poorly on the task (score <3 SD from the mean), therefore, their score was removed from the subsequent analysis. There was no evidence of a significant association between inattentive symptoms of ADHD and interoceptive sensitivity (*r* = 0.01, *p_s_* = 0.94), nor between interoceptive sensitivity and binge/disinhibited (*r* = −0.01, *p_s_* = 0.91) or restrictive eating (*r* = −0.07, *p_s_* = 0.40). Thus, no mediational analysis was conducted. Similarly interoceptive sensitivity scores were not significantly correlated with scores on the Reliance on Internal Hunger/Satiety Cues subscale of the Intuitive Eating Scale (*r* = −0.02, *p_s_* = 0.80).

## Discussion

The results of the two studies demonstrated that both the inattentive and hyperactive/impulsive symptoms of ADHD were associated with both binge/disinhibited and restrictive eating. In addition, in both studies negative mood, a composite index reflecting anxiety, depression and stress was a significant mediator of the association between core symptoms of ADHD and disordered eating. Furthermore, deficits in awareness and reliance on internal hunger/satiety signals were another mediator. However inattentive, but not hyperactive/impulsive, symptoms of ADHD were associated with lower levels of awareness and reliance on internal hunger/satiety cues, which in turn were associated with disordered eating, especially binge/disinhibited eating. In Study 2, interoceptive sensitivity assessed by a heartbeat task was found not to mediate the relationship between inattentive symptoms of ADHD and disordered eating. The results from both studies demonstrated that inattentive symptoms of ADHD directly related to binge/disinhibited eating behavior, while accounting for the indirect pathways of association *vi*a negative mood and awareness and reliance on internal hunger/satiety signals. In Study 2, hyperactive/impulsive symptoms of ADHD were also found to directly associate with both binge/disinhibited and restrictive eating (while controlling for the mediator variables). Impulsivity, as assessed *via* the BIS, directly predicted binge/disinhibited eating, but there was no evidence for a direct association with restrictive eating, which by implication suggests a specific contribution of impulsivity symptoms of ADHD to binge/disinhibited eating and hyperactivity symptoms to restrictive eating. Moderation analysis showed that none of the relationships between the core symptoms of ADHD and disordered eating were influenced by age, gender, ADHD medication, or BMI.

While there is strong consistent evidence for a positive association between ADHD and binge/disinhibited eating (8, 9, 65, 66), an association between ADHD and restrictive eating behavior has rarely been investigated in previous studies, and the findings have been inconsistent [for a review see Ref. (4)]. In this study, we used a composite index of restrictive eating which reflects a pattern of behavior to limit food intake as a means of controlling body weight, and found that ADHD symptoms, including both inattentive and hyperactive/impulsive symptoms, were positively associated with restrictive eating behavior in two independent samples.

Negative mood was a significant mediational pathway of association between core symptoms of ADHD and both binge/disinhibited and restrictive eating behavior. These findings suggest that anxiety, depression, and stress symptoms frequently reported in individuals with ADHD may provide an underlying mechanism for disordered eating behaviors. Schweickert et al. (31) suggested that compulsive eating in individuals with ADHD may be a compensatory mechanism to control frustration and anxiety associated with attention and organizational difficulties. In line with this hypothesis, Yates et al. (67) studied a population of inpatient women with EDs and found that depression scores were positively associated with current ADHD inattention symptoms and Nazar et al. (68) reported that depressive symptoms were the strongest predictor of binge eating in a sample of clinically obese women. In addition, stress and depression levels are predictive of binging behavior in both individuals with an eating disorder and a student population (69, 70). Similarly, restrictive eating may also be used as a coping mechanism for negative affect, and significant weight loss through extreme restrictive eating, as seen in AN, is another means of emotional regulation resulting in a short-term decrease in negative mood (71). These findings suggest that disordered eating, both binging and restrictive eating, may assist individuals with ADHD to control negative affect, especially in the absence of any other coping mechanisms. Our results extend the current state of knowledge by demonstrating how negative mood, potentially reflecting aspects of psychological distress, associated with ADHD symptoms (even at subclinical levels) can lead to eating pathology. This provides the first evidence for a mechanistic pathway of association between the core symptoms of ADHD and eating pathology *via* negative affect.

Decreased awareness of internal signals of hunger and satiety has been suggested as a possible pathway of association between inattentive symptoms of ADHD and disordered eating (8), but to the best of our knowledge this hypothesis had not been investigated in previous studies. The present results show that the inattentive symptoms of ADHD were associated with decreased awareness of internal signals of hunger and satiety, and in turn these deficits were positively associated with disordered eating, particularly binge/disinhibited eating. Decreased awareness and/or poor knowledge of internal hunger/satiety cues could indicate that individuals with ADHD primarily use external cues to guide their eating behavior. Avoidance of eating when hungry, in combination with decreased reliance on satiety signals, may foster overeating and explain an inability of individuals with ADHD to stop eating, especially in the presence of highly palatable foods.

We found no evidence of an association between inattentive symptoms of ADHD and interoceptive sensitivity as assessed by the heartbeat perception task. Similarly, we found that interoceptive sensitivity was not associated with disordered eating. Pollatos et al. (72) used a heartbeat perception task and found that patients with AN displayed significantly decreased interoceptive sensitivity compared to controls. However, more recently Eshkevari et al. (73) did not find any differences in a heartbeat detection task between individuals with EDs and healthy controls. These apparently contradictory findings suggest that further studies are required to investigate the use of a heartbeat task as an index of interoceptive sensitivity in individuals with EDs/disordered eating. In contrast to the report of Herbert et al. (47) we did not find that interoceptive sensitivity correlated with the Reliance on Internal Hunger/Satiety Cues subscale of the 21-item IES (46). This may suggest that the Reliance on Internal Hunger/Satiety Cues subscale indexes a distinct component of interoception from the heartbeat task, at least within our sample population. Specifically, the self-report questionnaire indexes both awareness and reliance on bodily signals *(hunger/satiety)*, whereas the heartbeat task measures somatic awareness only.

It is of particular interest that inattentive symptoms of ADHD directly predicted binge/disinhibited eating, independent of any influence of mood and awareness and reliance on internal hunger/satiety cues, suggesting that these pathways are not the sole mechanisms through which inattentive symptoms are associated with binge/disinhibited eating pathology. Several experimental studies have shown that distraction while eating (e.g., watching TV) affects the memory encoding of a meal and is associated with increased subsequent snack intake. Conversely, attentive eating has been found to enhance memory encoding and reduce later snack intake [for a review see Ref. (74)]. It is, therefore, plausible that individuals with pronounced inattentive symptoms of ADHD may be easily distracted when eating, resulting in impaired memory for recent eating, and subsequent overeating, especially in the presence of highly palatable foods. Further research is warranted to test this hypothesis.

In contrast to the findings from Study 1, the results from Study 2 provided evidence for a direct relationship between hyperactive/impulsive symptoms of ADHD and both binge/disinhibited eating and restrictive eating independent of the pathways *via* negative mood, and awareness and reliance on internal hunger/satiety cues. These findings may be attributed to differences in sample population characteristics and more specifically to the severity of ADHD symptoms. The Study 1 sample included individuals who reported a previous diagnosis of ADHD (33.3% of the total sample), whereas in Study 2, none of the participants reported a diagnosis of ADHD. Higher scores in the self-reported symptoms of ADHD suggest that participants in Study 1 had higher levels of ADHD symptoms than the participants in Study 2. It is, therefore, plausible that in individuals with lower levels of hyperactivity/impulsivity symptoms of ADHD, there are other pathways that may link their symptoms to disordered eating in addition to negative mood, which was found to be a significant mediator. However, at higher levels of hyperactivity/impulsivity no direct relationship is observed, and the effect of hyperactive/impulsive symptoms of ADHD on disordered eating is entirely mediated by negative mood. Interestingly, in Study 2 while impulsivity, as assessed by the BIS, directly predicted binge/disinhibited eating there was no evidence for a direct association with restrictive eating, thus, by implication, providing evidence for a specific contribution of impulsivity symptoms of ADHD to binge/disinhibited eating and hyperactivity symptoms of ADHD to restrictive eating. Further studies are warranted to replicate these initial findings.

To the best of our knowledge, no studies have examined the influence of age, BMI, and ADHD medication on the association between ADHD and disordered eating behavior using formal moderation analysis. In addition, only three previous studies conducted a formal moderation analysis to examine the effect of gender on the relationship between ADHD and disordered eating (75–77). Our findings are in agreement with the majority of previous evidence for a non-significant effect of gender on the association between ADHD symptomatology and binging behavior (76–81). Similarly, Alfonsson et al. (82) reported no significant gender effect on the association between ADHD and loss of control over eating, a core feature of binge eating. However, there is some limited evidence for a stronger association between the hyperactivity symptoms of ADHD and a drive for thinness in men than women (81, 83), which warrants further investigation.

Particular strengths of our studies are the relatively large sample sizes, the control for important confounds in the models and the simultaneous assessment of both binge/disinhibited eating, and restrictive eating behavior using well-validated measures both online and in a laboratory setting. The use of non-clinical samples ensures that confounds associated with clinical research in patients (e.g., medication status) can be minimized, providing a practical approach that allows mechanism testing. Furthermore, recruitment from the general population enables generalization of the findings to individuals in the population who experience symptoms of ADHD and EDs, but do not meet diagnostic criteria for the disorders.

Despite these strengths, there are certain limitations of our studies. First, the cross-sectional nature of the studies precludes causal inferences. For example, it cannot be excluded that the inattention symptoms may be secondary to malnutrition and eating pathology (84). In addition, despite the use of well-validated measures, most of the data collected in the present studies were based on self-reports, and it would, therefore, be valuable to validate these initial findings using objectively assessed measures. For example, the QbTest could be used to evaluate the core symptoms of ADHD (see https://www.qbtech.com/). In our studies, although we attempted to examine the specific contributions of hyperactive and impulsive symptoms of ADHD to disordered eating, we are aware that conclusions should be tempered, as no objective measure of hyperactivity was used. Furthermore, while findings from our studies suggest that core symptoms of ADHD relate both to binge/disinhibited and restrictive eating pathology, it remains unclear whether binge/disinhibited eating and restrictive eating characterize two distinct groups within our sample population or whether the core symptoms of ADHD predispose individuals to increased eating pathology, with cycling between binging and restrictive eating. To the best of our knowledge, no longitudinal study has examined the complex interaction that may arise due to cyclical shifting between different subtypes of EDs (85) and the change of predominant symptoms of ADHD that may occur during the lifetime (86). Our study is a valuable initial step toward elucidating the mediating factors that might explain the complex relationship between core symptoms of ADHD and disordered eating. Another potential issue for Study 1 is that internet-based data collection can result in selection or response bias, threatening the quality of web surveys (87). However, response rate was as high as 95.8% in the Study 1 online survey, suggesting that responders did not differ from non-responders. Furthermore, evidence suggests that questionnaire scores obtained online are comparable to those collected using traditional paper-and-pencil formats, and that psychometric properties of questionnaires are not adversely affected by computerized data collection (88). Indeed, it has been suggested that anonymous, web-based surveys may be more useful when collecting data on sensitive issues, such as eating- and body-related questions (89). Finally, despite the fact that the studies were advertised so that both males and females could take part, the sample in both studies comprised mainly female participants. There is also a potential sampling bias, as in both studies the participants were mainly highly educated adults. Future studies are warranted to replicate and extend the current findings in more representative and equally distributed samples of males and females. Given that participants in these studies were recruited from the general population, the results cannot be generalized to other population samples, such as adults with ADHD recruited from clinics and/or hospitalized patients with EDs, and future studies should also extend the present research to those populations.

The present findings suggest that prevention and intervention programs for eating pathology would be likely to benefit from treating mood disorders and interoception deficits in individuals who score highly on ADHD symptomatology. The direct relationship we identified between inattentive symptoms of ADHD and binge/disinhibited eating suggests that treatments and behavioral therapies that directly target attention deficits may be particularly effective in the management of both ADHD and binge eating disorder. Further, our findings of a possible association between ADHD and restrictive eating question whether the recently approved drug for the treatment of moderate to severe BED, lisdexamfetamine (90) would be an effective therapy for individuals with ADHD who exhibit restrictive eating. Ultimately, a better understanding of the range of specific eating problems experienced by individuals with ADHD and their underlying mechanisms will facilitate more effective and personalized treatment. Finally, as mood disorders are associated with the development of a range of psychopathologies including EDs (91), interventions targeting mood regulation may provide a transdiagnostic approach to resilience and mental health promotion programs and the prevention and treatment of psychopathology more broadly.

In conclusion, these studies showed that in two independent adult samples, ADHD symptoms were positively related to disordered eating, including both binge/disinhibited and restrictive eating and that negative mood mediated the relationships. Deficits in awareness and reliance on internal hunger/satiety signals provided another mechanistic pathway of association between inattentive symptoms of ADHD and disordered eating, especially binge/disinhibited eating. Notably, in both studies inattentive symptoms of ADHD were directly related to binge/disinhibited eating. These findings could have important implications for prevention and early intervention programs, which might usefully focus on mood regulation in individuals with ADHD symptoms at risk for developing disordered eating. Further investigation of the role of the inattentive symptoms of ADHD in disordered eating may be helpful in developing novel treatments for both ADHD and binge eating.

## Ethics Statement

This study was carried out in accordance with the recommendations of the Science, Technology, Engineering and Mathematics Ethical Review Committee, University of Birmingham, UK with online or written informed consent from all subjects. All subjects provided online or written informed consent in accordance with the Declaration of Helsinki. The protocol was approved by the Science, Technology, Engineering and Mathematics Ethical Review Committee, University of Birmingham, UK (Reference ERN_16-0538).

## Author Contributions

All authors were involved in the design of the study. PK collected the data and analyzed the results under supervision from SH, PR, and CD. The manuscript was written by PK with edits/comments provided by SH, PR, and CD. All authors contributed to and approved the final manuscript.

## Conflict of Interest Statement

CD is an employee, director, and shareholder of P1vital limited and a director and shareholder of P1vital products limited. All other authors declare no competing interests.

## References

[1] American Psychiatric Association. Diagnostic and Statistical Manual of Mental Disorders (DSM-5^®^). Washington: American Psychiatric Pub (2013).

[2] Biederman J Petty CR Clarke A Lomedico A Faraone SV Predictors of persistent ADHD: an 11-year follow-up study. J Psychiatr Res (2011) 45(2):150–5. 10.1016/j.jpsychires.2010.06.009 20656298 PMC3068747

[3] Instanes JT Klungsoyr K Halmoy A Fasmer OB Haavik J Adult ADHD and comorbid somatic disease: a systematic literature review. J Atten Disord (2016) 22(3):203–28. 10.1177/1087054716669589 27664125 PMC5987989

[4] Kaisari P Dourish CT Higgs S. Attention deficit hyperactivity disorder (ADHD) and disordered eating behaviour: a systematic review and a framework for future research. Clin Psychol Rev (2017) 53:109–21. 10.1016/j.cpr.2017.03.002 28334570

[5] Levin RL Rawana JS. Attention-deficit/hyperactivity disorder and eating disorders across the lifespan: a systematic review of the literature. Clin Psychol Rev (2016) 50:22–36. 10.1016/j.cpr.2016.09.010 27693587

[6] Nazar BP Bernardes C Peachey G Sergeant J Mattos P Treasure J. The risk of eating disorders comorbid with attention-deficit/hyperactivity disorder: a systematic review and meta-analysis. Int J Eat Disord (2016) 49(12):1045–57. 10.1002/eat.22643 27859581

[7] Reinblatt SP. Are eating disorders related to attention deficit/hyperactivity disorder? Curr Treat Options Psychiatry (2015) 2(4):402–12. 10.1007/s40501-015-0060-7 26949595 PMC4777329

[8] Davis C Levitan RD Smith M Tweed S Curtis C. Associations among overeating, overweight, and attention deficit/hyperactivity disorder: a structural equation modelling approach. Eat Behav (2006) 7(3):266–74. 10.1016/j.eatbeh.2005.09.006 16843230

[9] Strimas R Davis C Patte K Curtis C Reid C McCool C. Symptoms of attention-deficit/hyperactivity disorder, overeating, and body mass index in men. Eat Behav (2008) 9(4):516–8. 10.1016/j.eatbeh.2008.07.005 18928919

[10] Tong L Shi H Li X. Associations among ADHD, abnormal eating and overweight in a non-clinical sample of Asian children. Sci Rep (2017) 7(1):2844. 10.1038/s41598-017-03074-4 28588278 PMC5460237

[11] Yilmaz Z Javaras KN Baker JH Thornton LM Lichtenstein P Bulik CM Association between childhood to adolescent attention deficit/hyperactivity disorder symptom trajectories and late adolescent disordered eating. J Adolesc Health (2017) 61(2):140–6. 10.1016/j.jadohealth.2017.04.001 28734322 PMC5726271

[12] Mikami AY Hinshaw SP Arnold LE Hoza B Hechtman L Newcorn JH Bulimia nervosa symptoms in the multimodal treatment study of children with ADHD. Int J Eat Disord (2010) 43(3):248–59. 10.1002/eat.20692 19378318

[13] Mikami AY Hinshaw SP Patterson KA Lee JC. Eating pathology among adolescent girls with attention-deficit/hyperactivity disorder. J Abnorm Psychol (2008) 117(1):225–35. 10.1037/0021-843x.117.1.225 18266500 PMC2930179

[14] Muller A Claes L Wilderjans TF de Zwaan M. Temperament subtypes in treatment seeking obese individuals: a latent profile analysis. Eur Eat Disord Rev (2014) 22(4):260–6. 10.1002/erv.2294 24809764

[15] Seitz J Kahraman-Lanzerath B Legenbauer T Sarrar L Herpertz S Salbach-Andrae H The role of impulsivity, inattention and comorbid ADHD in patients with bulimia nervosa. PLoS One (2013) 8(5):e63891. 10.1371/journal.pone.0063891 23700439 PMC3659086

[16] Nagl M Jacobi C Paul M Beesdo-Baum K Hofler M Lieb R Prevalence, incidence, and natural course of anorexia and bulimia nervosa among adolescents and young adults. Eur Child Adolesc Psychiatry (2016) 25(8):903–18. 10.1007/s00787-015-0808-z 26754944

[17] Goldschmidt AB Aspen VP Sinton MM Tanofsky-Kraff M Wilfley DE Disordered eating attitudes and behaviors in overweight youth. Obesity (Silver Spring) (2008) 16(2):257–64. 10.1038/oby.2007.48 18239631

[18] Claridge G Davis C Personality and Psychological Disorders. New York: Routledge (2013).

[19] Insel T Cuthbert B Garvey M Heinssen R Pine DS Quinn K Research domain criteria (RDoC): toward a new classification framework for research on mental disorders. Am J Psychiatry (2010) 167(7):748–51. 10.1176/appi.ajp.2010.09091379 20595427

[20] Friedrichs B Igl W Larsson H Larsson JO Coexisting psychiatric problems and stressful life events in adults with symptoms of ADHD – a large Swedish population-based study of twins. J Atten Disord (2012) 16(1):13–22. 10.1177/1087054710376909 20686099

[21] Hodgkins P Montejano L Sasane R Huse D. Cost of illness and comorbidities in adults diagnosed with attention-deficit/hyperactivity disorder: a retrospective analysis. Prim Care Companion CNS Disord (2011) 13(2):CC.10m01030. 10.4088/PCC.10m01030 21977356 PMC3184593

[22] Rucklidge JJ Downs-Woolley M Taylor M Brown JA Harrow SE. Psychiatric comorbidities in a New Zealand sample of adults with ADHD. J Atten Disord (2016) 20(12):1030–8. 10.1177/1087054714529457 24743977

[23] Uchida M Spencer TJ Faraone SV Biederman J Adult outcome of ADHD: an overview of results from the MGH longitudinal family studies of pediatrically and psychiatrically referred youth with and without ADHD of both sexes. J Atten Disord (2018) 22(6):523–34. 10.1177/1087054715604360 26396145

[24] Hirvikoski T Lindholm T Nordenstrom A Nordstrom AL Lajic S. High self-perceived stress and many stressors, but normal diurnal cortisol rhythm, in adults with ADHD (attention-deficit/hyperactivity disorder). Horm Behav (2009) 55(3):418–24. 10.1016/j.yhbeh.2008.12.004 19162030

[25] Linardon J Wade T Psychotherapy for bulimia nervosa on symptoms of depression: a meta-analysis of randomized controlled trials. Int J Eat Disord (2017) 50(10):1124–36. 10.1002/eat.22763 28804915

[26] Spindler A Milos G. Links between eating disorder symptom severity and psychiatric comorbidity. Eat Behav (2007) 8(3):364–73. 10.1016/j.eatbeh.2006.11.012 17606234

[27] Swinbourne J Hunt C Abbott M Russell J St Clare T Touyz S. The comorbidity between eating disorders and anxiety disorders: prevalence in an eating disorder sample and anxiety disorder sample. Aust N Z J Psychiatry (2012) 46(2):118–31. 10.1177/0004867411432071 22311528

[28] Biederman J Newcorn J Sprich S. Comorbidity of attention deficit hyperactivity disorder with conduct, depressive, anxiety, and other disorders. Am J Psychiatry (1991) 148(5):564–77. 10.1176/ajp.148.5.564 2018156

[29] Quinn PO. Attention-deficit/hyperactivity disorder and its comorbidities in women and girls: an evolving picture. Curr Psychiatry Rep (2008) 10(5):419–23. 10.1007/s11920-008-0067-5 18803916

[30] Ziobrowski H Brewerton TD Duncan AE. Associations between ADHD and eating disorders in relation to comorbid psychiatric disorders in a nationally representative sample. Psychiatry Res (2017) 260:53–9. 10.1016/j.psychres.2017.11.026 29172099

[31] Schweickert LA Strober M Moskowitz A. Efficacy of methylphenidate in bulimia nervosa comorbid with attention-deficit hyperactivity disorder: a case report. Int J Eat Disord (1997) 21(3):299–301. 10.1002/(SICI)1098-108X(199704)21:3<299::AID-EAT11>3.0.CO;2-W 9097204

[32] Fleming J Levy L Eating disorders in women with AD/HD. In: Quinn PO Nadeau KG, editors. Gender Issues and AD/HD: Research, Diagnosis and Treatment. Silver Springs, MD: Advantage Books (2002). p. 411–26.

[33] Avalos LC Tylka TL Exploring a model of intuitive eating with college women. J Couns Psychol (2006) 53(4):486 10.1037/0022-0167.53.4.486

[34] Fritz MS MacKinnon DP. Required sample size to detect the mediated effect. Psychol Sci (2007) 18(3):233–9. 10.1111/j.1467-9280.2007.01882.x 17444920 PMC2843527

[35] Meade AW Craig SB. Identifying careless responses in survey data. Psychol Methods (2012) 17(3):437. 10.1037/a0028085 22506584

[36] Conners CK Erhardt D Sparrow E CAARS: Conner’s Adult ADHD Rating Scales. North Tonawanda, NY: Multi-Health Systems Incorporated (MHS) (1999).

[37] Ghassemi F Moradi MH Tehrani-Doost M Abootalebi V. Evaluation of estimating missed answers in conners adult ADHD rating scale (screening version). Iran J Psychiatry (2010) 5(3):108–12. 22952502 PMC3430501

[38] Van Strien T Frijters JE Bergers G Defares PB The Dutch eating behavior questionnaire (DEBQ) for assessment of restrained, emotional, and external eating behavior. Int J Eat Disord (1986) 5(2):295–315. 10.1002/1098-108X(198602)5:2<295::AID-EAT2260050209>3.0.CO;2-T

[39] Latner JD Mond JM Kelly MC Haynes SN Hay PJ. The loss of control over eating scale: development and psychometric evaluation. Int J Eat Disord (2014) 47(6):647–59. 10.1002/eat.22296 24862351

[40] Gormally J Black S Daston S Rardin D. The assessment of binge eating severity among obese persons. Addict Behav (1982) 7(1):47–55. 10.1016/0306-4603(82)90024-7 7080884

[41] Timmerman GM Binge eating scale: further assessment of validity and reliability. J Appl Biobehav Res (1999) 4(1):1–12. 10.1111/j.1751-9861.1999.tb00051.x

[42] Henderson M Freeman CP A self-rating scale for bulimia. The’BITE’.Br J Psychiatry (1987) 150(1):18–24. 10.1192/bjp.150.1.18 3651670

[43] Garner DM Olmsted MP Bohr Y Garfinkel PE. The eating attitudes test: psychometric features and clinical correlates. Psychol Med (1982) 12(04):871–8. 10.1017/S0033291700049163 6961471

[44] Anstine D Grinenko D. Rapid screening for disordered eating in college-aged females in the primary care setting. J Adolesc Health (2000) 26(5):338–42. 10.1016/S1054-139X(99)00120-2 10775826

[45] Mintz LB O’Halloran MS. The eating attitudes test: validation with DSM-IV eating disorder criteria. J Pers Assess (2000) 74(3):489–503. 10.1207/S15327752JPA7403_11 10900574

[46] Tylka TL Development and psychometric evaluation of a measure of intuitive eating. J Couns Psychol (2006) 53(2):226 10.1037/0022-0167.53.2.226

[47] Herbert BM Blechert J Hautzinger M Matthias E Herbert C. Intuitive eating is associated with interoceptive sensitivity. Effects on body mass index. Appetite (2013) 70:22–30. 10.1016/j.appet.2013.06.082 23811348

[48] Zigmond AS Snaith RP. The hospital anxiety and depression scale. Acta Psychiatr Scand (1983) 67(6):361–70. 10.1111/j.1600-0447.1983.tb09716.x 6880820

[49] Bjelland I Dahl AA Haug TT Neckelmann D The validity of the hospital anxiety and depression scale: an updated literature review. J Psychosom Res (2002) 52(2): 69–77. 11832252 10.1016/s0022-3999(01)00296-3

[50] Cohen S Kamarck T Mermelstein R A global measure of perceived stress. J Health Soc Behav (1983) 24(4):385–96. 10.2307/2136404 6668417

[51] Leung D Lam T Chan S Three versions of the perceived stress scale: validation in a sample of Chinese cardiac patients who smoke. BMC Public Health (2010) 10:513 10.1186/1471-2458-10-513 20735860 PMC2939644

[52] Mimura C Griffiths P. A Japanese version of the perceived stress scale: cross-cultural translation and equivalence assessment. BMC Psychiatry (2008) 8(1):1. 10.1186/1471-244X-8-85 18826581 PMC2569029

[53] Selzer ML Vinokur A van Rooijen L A self-administered short Michigan alcoholism screening test (SMAST). J Stud Alcohol (1975) 36(1):117–26. 10.15288/jsa.1975.36.117 238068

[54] Skinner HA. The drug abuse screening test. Addict Behav (1982) 7(4):363–71. 10.1016/0306-4603(82)90005-3 7183189

[55] Yudko E Lozhkina O Fouts A. A comprehensive review of the psychometric properties of the drug abuse screening test. J Subst Abuse Treat (2007) 32(2):189–98. 10.1016/j.jsat.2006.08.002 17306727

[56] Hayes AF Introduction to Mediation, Moderation, and Conditional Process Analysis: A Regression-Based Approach. New York: Guilford Press (2013).

[57] Howell DC Statistical Methods Psychology. International Edition: Cengage Learning (2013).

[58] Herbert BM Muth ER Pollatos O Herbert C. Interoception across modalities: on the relationship between cardiac awareness and the sensitivity for gastric functions. PLoS One (2012) 7(5):e36646. 10.1371/journal.pone.0036646 22606278 PMC3350494

[59] Whitehead WE Drescher VM Perception of gastric contractions and self-control of gastric motility. Psychophysiology (1980) 17(6):552–8. 10.1111/j.1469-8986.1980.tb02296.x 7443922

[60] Herbert BM Pollatos O. The body in the mind: on the relationship between interoception and embodiment. Top Cogn Sci (2012) 4(4):692–704. 10.1111/j.1756-8765.2012.01189.x 22389201

[61] Herbert BM Herbert C Pollatos O Weimer K Enck P Sauer H Effects of short-term food deprivation on interoceptive awareness, feelings and autonomic cardiac activity. Biol Psychol (2012) 89(1):71–9. 10.1016/j.biopsycho.2011.09.004 21958594

[62] Patton JH Stanford MS Barratt ES. Factor structure of the Barratt impulsiveness scale. J Clin Psychol (1995) 51(6):768–74. 10.1002/1097-4679(199511)51:6<768::AID-JCLP2270510607>3.0.CO;2-1 8778124

[63] Stanford MS Mathias CW Dougherty DM Lake SL Anderson NE Patton JH Fifty years of the Barratt impulsiveness scale: an update and review. Pers Individ Dif (2009) 47(5):385–95. 10.1016/j.paid.2009.04.008

[64] Schandry R Heart beat perception and emotional experience. Psychophysiology (1981) 18(4):483–8. 10.1111/j.1469-8986.1981.tb02486.x 7267933

[65] Hudson JI Hiripi E Pope HG Jr Kessler RC. The prevalence and correlates of eating disorders in the national comorbidity survey replication. Biol Psychiatry (2007) 61(3):348–58. 10.1016/j.biopsych.2006.03.040 16815322 PMC1892232

[66] Swanson SA Crow SJ Le Grange D Swendsen J Merikangas KR. Prevalence and correlates of eating disorders in adolescents. Results from the national comorbidity survey replication adolescent supplement. Arch Gen Psychiatry (2011) 68(7):714–23. 10.1001/archgenpsychiatry.2011.22 21383252 PMC5546800

[67] Yates WR Lund BC Johnson C Mitchell J McKee P. Attention-deficit hyperactivity symptoms and disorder in eating disorder inpatients. Int J Eat Disord (2009) 42(4):375–8. 10.1002/eat.20627 19040267

[68] Nazar BP Suwwan R de Sousa Pinna CM Duchesne M Freitas SR Sergeant J Influence of attention-deficit/hyperactivity disorder on binge eating behaviors and psychiatric comorbidity profile of obese women. Compr Psychiatry (2014) 55(3):572–8. 10.1016/j.comppsych.2013.09.015 24246603

[69] Greenberg BR Predictors of binge eating in bulimic and nonbulimic women. Int J Eat Disord (1986) 5(2):269–84. 10.1002/1098-108X(198602)5:2<269::AID-EAT2260050207>3.0.CO;2-3

[70] Stice E. Risk and maintenance factors for eating pathology: a meta-analytic review. Psychol Bull (2002) 128(5):825. 10.1037/0033-2909.128.5.825 12206196

[71] Fairburn CG Shafran R Cooper Z. A cognitive behavioural theory of anorexia nervosa. Behav Res Ther (1999) 37(1):1–13. 10.1016/S0005-7967(98)00102-8 9922553

[72] Pollatos O Kurz AL Albrecht J Schreder T Kleemann AM Schopf V Reduced perception of bodily signals in anorexia nervosa. Eat Behav (2008) 9(4):381–8. 10.1016/j.eatbeh.2008.02.001 18928900

[73] Eshkevari E Rieger E Musiat P Treasure J. An investigation of interoceptive sensitivity in eating disorders using a heartbeat detection task and a self-report measure. Eur Eat Disord Rev (2014) 22(5):383–8. 10.1002/erv.2305 24985151

[74] Higgs S. Cognitive processing of food rewards. Appetite (2016) 104:10–7. 10.1016/j.appet.2015.10.003 26458961

[75] Bleck J DeBate RD. Exploring the co-morbidity of attention-deficit/hyperactivity disorder with eating disorders and disordered eating behaviors in a nationally representative community-based sample. Eat Behav (2013) 14(3):390–3. 10.1016/j.eatbeh.2013.05.009 23910787

[76] Davis C Cohen A Davids M Rabindranath A. Attention-deficit/hyperactivity disorder in relation to addictive behaviors: a moderated-mediation analysis of personality-risk factors and sex. Front Psychiatry (2015) 6:47. 10.3389/fpsyt.2015.00047 25941494 PMC4403287

[77] Patte KA Davis CA Levitan RD Kaplan AS Carter-Major J Kennedy JL. A behavioral genetic model of the mechanisms underlying the link between obesity and symptoms of ADHD. J Atten Disord (2016):1087054715618793. 10.1177/1087054715618793 26794671

[78] Brewerton TD Duncan AE. Associations between attention deficit hyperactivity disorder and eating disorders by gender: results from the national comorbidity survey replication. Eur Eat Disord Rev (2016) 24(6):536–40. 10.1002/erv.2468 27480884

[79] Mattos P Saboya E Ayrao V Segenreich D Duchesne M Coutinho G Comorbid eating disorders in a Brazilian attention-deficit/hyperactivity disorder adult clinical sample. Rev Bras Psiquiatr (2004) 26(4):248–50. 10.1590/S1516-44462004000400008 15729458

[80] Pauli-Pott U Becker K Albayrak O Hebebrand J Pott W Links between psychopathological symptoms and disordered eating behaviors in overweight/obese youths. [Erratum appears in Int J Eat Disord. 2014 Jul;47(5):563]. Int J Eat Disord (2013) 46(2):156–63. 10.1002/eat.22055 22987501

[81] Slane JD Burt SA Klump KL. The road less traveled: associations between externalizing behaviors and eating pathology. Int J Eat Disord (2010) 43(2):149–60. 10.1002/eat.20680 19350646

[82] Alfonsson S Parling T Ghaderi A Self‐reported symptoms of adult attention deficit hyperactivity disorder among obese patients seeking bariatric surgery and its relation to alcohol consumption, disordered eating and gender. Clin Obes (2013) 3(5):124–31. 25586627 10.1111/cob.12025

[83] Grabarek C Cooper S. Graduate students’ social and emotional functioning relative to characteristics of eating disorders. J Gen Psychol (2008) 135(4):425–51. 10.3200/GENP.135.4.425-452 18959231

[84] Lauer CJ Gorzewski B Gerlinghoff M Backmund H Zihl J. Neuropsychological assessments before and after treatment in patients with anorexia nervosa and bulimia nervosa. J Psychiatr Res (1999) 33(2):129–38. 10.1016/S0022-3956(98)00020-X 10221745

[85] Fairburn CG Harrison PJ. Eating disorders. Lancet (2003) 361(9355):407–16. 10.1016/s0140-6736(03)12378-1 12573387

[86] Holbrook JR Cuffe SP Cai B Visser SN Forthofer MS Bottai M Persistence of parent-reported ADHD symptoms from childhood through adolescence in a community sample. J Atten Disord (2016) 20(1):11–20. 10.1177/1087054714539997 24994874 PMC4474771

[87] Couper M Web surveys: a review of issues and approaches. Public Opin Q (2000) 64(4):464–94. 10.1086/318641 11171027

[88] Naus MJ Philipp LM Samsi M From paper to pixels: a comparison of paper and computer formats in psychological assessment. Comput Human Behav (2009) 25(1):1–7. 10.1016/j.chb.2008.05.012

[89] Kays K Gathercoal K Buhrow W Does survey format influence self-disclosure on sensitive question items? Comput Human Behav (2012) 28(1):251–6. 10.1016/j.chb.2011.09.007

[90] US Food and Drug Administration. FDA expands uses of Vyvanse to treat binge-eating disorder US Food and Drug Administration (2015). Available from: http://web.archive.org/web/20180126103215/http://www.fda.gov/newsevents/newsroom/pressannouncements/ucm432543.htm (Accessed: March 19, 2018).

[91] Casper RC Depression and eating disorders. Depress Anxiety (1998) 8(S1):96–104. 10.1002/(SICI)1520-6394(1998)8:1+<96::AID-DA15>3.0.CO;2-4 9809221

