# Supplementary material for: Associations Between Core Symptoms of Attention Deficit Hyperactivity Disorder and Both Binge and Restrictive Eating
Source: Front Psychiatry. 2018 Mar 29;9:103. doi: 10.3389/fpsyt.2018.00103 (PMC5884932; doi:10.3389/fpsyt.2018.00103)
Supplement: Supplementary file 1 [file data_sheet_1.PDF]

Supplementary Material

Associations Between Core Symptoms of Attention Deficit Hyperactivity Disorder (ADHD) and Both Binge and Restrictive Eating

Panagiota Kaisari*, Colin T Dourish, Pia Rotshtein and Suzanne Higgs

***Corresponding author:** P.Kaisari.1@bham.ac.uk

Table S1 Component loadings for principal component analysis of disordered eating variables.

	Component 1: Binge/Disinhibited Eating	Component 2: Restrictive Eating
BITE	0.929	
BES	0.926	
LOCES	0.897	
DEBQ-Emotional Eating	0.830	
DEBQ-External Eating	0.739	
EAT-Bulimia & Food Preoccupation	0.725	
EAT-Oral Control		0.728
EAT-Dieting		0.703
DEBQ-Restraint Eating		0.532

Table S2 Cronbach's alpha values for the questionnaire measures in Study 2.

**Measures**	**Cronbach's alpha**
Disordered Eating	
DEBQ - Emotional eating	0.96
DEBQ - External eating	0.86
DEBQ - Dietary Restraint	0.94
LOCES	0.93
BES	0.91
BITE	0.89
EAT Total score	0.91
EAT - Dieting	0.88
EAT - Bulimia	0.86
EAT - Oral Control	0.68
Awareness and reliance on internal hunger/satiety cues	
RIH	0.78
Negative mood	
HADS - Anxiety	0.82

Table S2 Continued

**Measures**	**Cronbach's alpha**
HADS - Depression	0.75
PSS	0.87
Covariates	
SMAST [£]	0.51
DAST-10	0.46

[£] Based on total 9 scale items. Items 8, 10, 12 and 13 had zero variance and were removed.

Go/No-Go Task: Both the neutral and food-based go/no-go tasks were programmed using E-Prime 2.0 software (Psychology Software tools, Inc., Sharpsburg, PA, USA; see Figure S1). In the neutral task, sports equipment and toiletries images were used; there were two conditions, with the sports equipment images comprising the ‘’go’’ stimuli in one condition (toiletries images: ‘’no go’’ stimuli) and the ‘’no go’’ stimuli in the other condition (toiletries images: ‘’ go’’ stimuli). In the food-based task, high-energy dense food and low-energy dense food images were used. Similarly, to the neutral task there were two conditions, with the high-energy dense food images comprising the ‘’go’’ stimuli in one condition (low-energy dense food images: ‘’no go’’ stimuli) and the ‘’no go’’ stimuli in the other condition (low-energy dense food images: ‘’ go’’ stimuli). The stimuli were selected from the BOSS database of normative photographs of objects (1). The BOSS images are normalised for category, familiarity, visual complexity, object agreement and viewpoint agreement, making them equivalent in valence and perceptual characteristics (1). All participants completed both tasks. The four conditions were presented in a randomised order (See Figure S1 for details of the task). The measure of interest was the number of commission errors (responses incorrectly made in ‘no-go' trials).

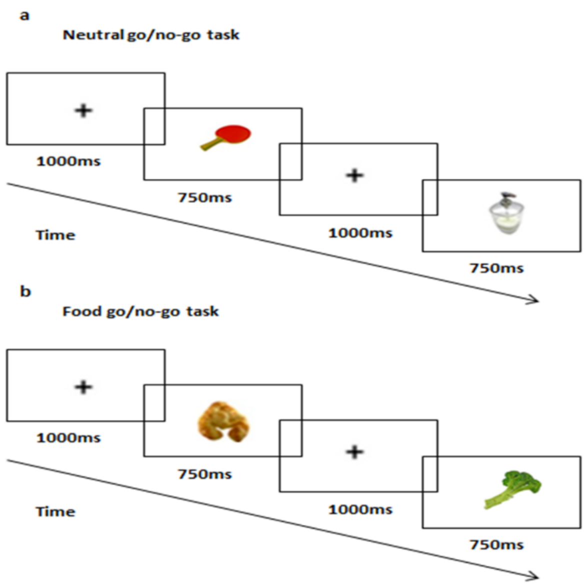

Figure S1 Go/no-go task: A total of 320 trials were presented, 160 (in two blocks of 80; 20 trials/condition) for the neutral task (**a**) and the same for the food task (**b**). Images were presented using a ratio of 80% ‘go' to 20% ‘no-go' trials to create a prepotent ‘go' response. Each trial was presented for 750 ms and was preceded by a fixation point for 1000 ms. The go and no-go categories were presented in a randomised order. Presentation order of the food and neutral tasks was randomised.

REFERENCES

1. Brodeur MB, Dionne-Dostie E, Montreuil T, Lepage M. The bank of standardized stimuli (BOSS), a new set of 480 normative photos of objects to be used as visual stimuli in cognitive research. PLoS One (2010) 5(5):e10773. doi:10.1371/journal.pone.0010773
